# Dynamic causal modelling of effective connectivity from fMRI: Are results reproducible and sensitive to Parkinson's disease and its treatment?

**DOI:** 10.1016/j.neuroimage.2009.12.080

**Published:** 2010-09

**Authors:** J.B. Rowe, L.E. Hughes, R.A. Barker, A.M. Owen

**Affiliations:** a University of Cambridge Department of Clinical Neurosciences, CB2 2QQ, UK; b Medical Research Council Cognition and Brain Sciences Unit, Cambridge, CB2 7EF, UK; c University of Cambridge Behavioural and Clinical Neuroscience Institute, CB2 3EB, UK; d Cambridge Centre for Brain Repair, CB2 OPY, UK

## Abstract

Dynamic causal modelling (DCM) of functional magnetic resonance imaging (fMRI) data offers new insights into the pathophysiology of neurological disease and mechanisms of effective therapies. Current applications can be used both to identify the most likely functional brain network underlying observed data and estimate the networks' connectivity parameters. We examined the reproducibility of DCM in healthy subjects (young 18–48 years, *n* = 27; old 50–80 years, *n* = 15) in the context of action selection. We then examined the effects of Parkinson's disease (50–78 years, Hoehn and Yahr stage 1–2.5, *n* = 16) and dopaminergic therapy. Forty-eight models were compared, for each of 90 sessions from 58 subjects. Model-evidences clustered according to sets of structurally similar models, with high correlations over two sessions in healthy older subjects. The same model was identified as most likely in healthy controls on both sessions and in medicated patients. In this most likely network model, the selection of action was associated with enhanced coupling between prefrontal cortex and the pre-supplementary motor area. However, the parameters for intrinsic connectivity and contextual modulation in this model were poorly correlated across sessions. A different model was identified in patients with Parkinson's disease after medication withdrawal. In “off” patients, action selection was associated with enhanced connectivity from prefrontal to lateral premotor cortex. This accords with independent evidence of a dopamine-dependent functional disconnection of the SMA in Parkinson's disease. Together, these results suggest that DCM model selection is robust and sensitive enough to study clinical populations and their pharmacological treatment. For critical inferences, model selection may be sufficient. However, caution is required when comparing groups or drug effects in terms of the connectivity parameter estimates, if there are significant posterior covariances among parameters.

## Introduction

The advent of formal analytic methods to assess connectivity in functional brain networks has enabled a much richer approach to the understanding of the pathophysiology of disease, and the neurobiological basis of effective therapies or recovery. These applications include neurodevelopmental and neuropsychiatric disorders (Kim et al., 2008; Stephan et al., 2006), healthy aging (Grady et al., 2003; Rowe et al., 2006) neurodegenerative disease (Grafton et al., 1994; Rowe et al., 2002b; Sonty et al., 2007) and real or simulated focal lesions (Grefkes et al., 2008; Kim and Horwitz, 2009; Rowe et al., 2007; Sharma et al., 2009). Indeed, these studies have often observed that measures of network connectivity are more sensitive to pathophysiology or treatment than standard analysis of regional activations. Increasing applications of these methods in clinical and non-clinical settings are therefore likely.

Dynamic causal modelling (DCM) has aroused particular interest since its introduction, in part because of the underlying Bayesian framework that permits model comparison among both nested and non-nested models (Friston et al., 2003; Penny et al., 2004a; Stephan et al., 2009a). The advantages of DCM have been discussed elsewhere (Penny et al., 2004b) with some direct empirical comparisons with other methods (Passamonti et al., 2008). However, future applications to clinical and pharmacological studies will be strengthened by information about the ability of DCM to re-identify evidence based network models as well as the ability to replicate model parameters (Schuyler et al., 2009).

In this paper, we address several issues that are critical if DCM is used to study clinical states or the effects of pharmacological interventions on network connectivity. In particular, (1) do DCM based model comparison procedures re-identify the same network model as “most likely” given evidence from data acquired in different sessions from the same individuals? Stability of model selection might be affected by day to day variations in cognitive states or technical factors like varying signal to noise and the BOLD signal. These factors are also important for classical analysis of regional activations across different sessions, including pharmacological fMRI (Iannetti and Wise, 2007; Lund et al., 2005; McGonigle et al., 2000). (2) Does DCM model selection identify differences in the network dynamics of a patient group? If so, does this support independent evidence for dysfunction of one or more elements of that network? While DCM will give new insights into network dynamics, there are areas of potential concordance between DCM and other methods such as analysis of regional activations, which could be identified. (3) Within the selected network model, does the application of DCM to data across different sessions replicate the parameter estimates for intrinsic connectivity and modulatory effects of the task context as expressed by the bilinear terms? Evidence of replication within-subjects across-sessions would support the use of DCM to explore between group differences in terms of connectivity parameters. Preliminary results are encouraging with high reliability in simple models (Schuyler et al., 2009). (4) Finally, is DCM sensitive to an effective intervention, such as an established pharmacological treatment in a defined clinical population?

To answer these questions empirically, we assessed DCM in the context of action selection (Rowe et al., 2005) by young and older healthy adults and older adults with the common neurodegenerative movement disorder, Parkinson's disease. We chose the action-selection paradigm because it gives robust fMRI activations of a reproducible set of distributed cortical activations in diverse populations (Deiber et al., 1991; Forstmann et al., 2008; Rowe et al., 2008a, 2005). Moreover, the paradigm is *prima facie* relevant to Parkinson's disease. In addition, there is an established literature with other methods to assess effective connectivity in the motor system with Parkinson's disease, including structural equation modelling (SEM) of fMRI (Palmer et al., 2009; Rowe et al., 2002b), path analysis of positron emission tomography data (Grafton et al., 1994) and regression models for fMRI embodying psychophysiological interactions (PPI) (Helmich et al., 2009, 2007).

Studies of Parkinson's disease using analyses of connectivity (SEM, PPI) or regional activations (voxelwise general linear models) have suggested a shift away from a medial route to voluntary action (from prefrontal cortex via pre-SMA and SMA to motor cortex) towards a lateral route (via lateral premotor cortex). This is partly driven by primary dysfunction of the supplementary motor area (SMA) and pre-SMA (Haslinger et al., 2001; Jahanshahi et al., 1995; Playford et al., 1992; Sabatini et al., 2000; Samuel et al., 1997; Yu et al., 2007) or a functional disconnection of the SMA (Rowe et al., 2002b), with compensatory enhancement of lateral premotor systems (the deficit driving the shift may be downstream of the dysfunctional nigrostriatal pathway output to the cortex via the thalamus, or reflect changes intrinsic to the SMA/pre-SMA). Moreover, this dysfunction of the pre-SMA and SMA is responsive to dopaminergic therapy (Buhmann et al., 2003; Haslinger et al., 2001; Jenkins et al., 1992; Rascol et al., 1994) and accentuated for self-generated or chosen actions, in comparison with specified or cued actions. This disease and therapeutic model is therefore well suited to test the performance of DCM, provided that good practice recommendations are adhered to (Stephan et al., 2009b).

Our first hypothesis was that DCM is replicable across sessions. We took a hierarchical approach, first considering the replication of model selection and then the reliability of parameter estimates within the most likely model. Through systematic variations on a prior structural model, we tested secondary hypotheses about feed-forward and feed-back connections; direct and modulatory effects of task manipulations; and the likely site of task related changes in effective connectivity. We also predicted that DCM can identify changes in motor networks in Parkinson's disease that reflect a shift away from the medial route to action (via SMA and pre-SMA) towards a lateral route (via lateral premotor cortex) for voluntary action. Finally, we predicted that this connectivity change in PD is sensitive to dopaminergic therapy.

## Methods

### Subjects and task

Sixteen patients (PD, 50–80 years) with idiopathic Parkinson's disease were recruited from the Cambridge Centre for Brain Repair's PD research clinic, using the UK PD Brain Bank clinical diagnostic criteria. Patients were tested once after their usual dopaminergic medication and once after dopaminergic mediation had been withdrawn (minimum 12 h for short acting preparations, 24 h for long acting preparations such as modified/slow-release preparations of l-dopa and carbergoline). The order of “on” vs. “off” sessions was randomly permuted within blocks of six successive subjects. Patients were examined on both occasions using the UPDRS-III motor rating scale (Fahn et al., 1987) and staged with the Hoehn and Yahr scale (Hoehn and Yahr, 1967).

Fifteen healthy older controls subjects (OC, 50–80 years) and twenty-eight healthy younger adults (18 to 48 years) were recruited from the PD research clinic database and the volunteer panel of the MRC Cognition and Brain Sciences Unit. Older subjects participated twice in the study, and their two sessions were randomly assigned to sessions A and B, similar to patient assignment to ‘on’ vs. ‘off’ sessions. The younger subjects participated once only. All subjects were right handed with no current depressive illness, no dementia based on prior cognitive assessment and no significant neurological (except Parkinson's disease) or psychiatric illness. The study was given a favourable opinion by the local Research Ethics Committee and participants gave written informed consent according to the 1991 Declaration of Helsinki. Subject details are summarised in supplementary Table S1.

A simple finger-tapping task used visually paced right hand button presses, in which subjects were presented with a picture of a right hand and pressed a button with one of their four right hand fingers in response to a cue. A small empty circle was presented above each finger. Half of responses were “specified,” by a single circle changing colour to black for 1 s, indicating which finger to press. Half of responses were “chosen,” indicated by all four circles changing to black for 1 s during which subjects could choose which of the four fingers to press. For this condition subjects were asked to make a “fresh choice on each trial regardless of what you have pressed before.” This might ideally lead one to make random choices. However, they were specifically not asked to make random selections as this can paradoxically invoke very non-random choices. Subjects made 40 specified responses and 40 chosen responses, interspersed with 40 null events (no change of colour in the circles above each finger, appearing to the subjects the same as the interstimulus interval cue). Cues were presented for 1 s with a stimulus onset asynchrony of 2.5 s, randomly intermixed, under control from Cogent 2000 software (www.vislab.ucl.ac.uk/Cogent2000) using Matlab 7.1 (www.mathworks.com) in Windows XP (www.microsoft.com).

### MRI data acquisition and analysis of regional activations

A Siemens Tim Trio 3 Tesla scanner was used to acquire BOLD sensitive T2⁎ weighted EPI images (TR 2000 ms, TE 30 ms, FA 78 degrees) with 32 slices, 3.0 mm thick, in-plane resolution 3 × 3 mm, with slice separation 0.75 mm, in sequential descending order. 156 volumes were acquired, the first 6 of which were discarded to allow for steady-state magnetisation. An MPRAGE T1-weighted structural image was also acquired for each subject (TR 2250 ms, TE 2.99 ms, FA 9 degrees, IT 900 ms, 256 × 256 × 192 isotropic 1 mm voxels).

Data pre-processing and analysis used SPM5 (http://www.fil.ion.ucl.ac.uk /spm) in Matlab 7.1 (R14, Mathworks, CA). fMRI data were converted from DICOM to NIFTII format, spatially realigned to the first image and sinc interpolated in time to the middle slice time to correct for acquisition delay. The mean fMRI volume and MPRAGE were coregistered using mutual information, and the MPRAGE segmented and normalised to the Montreal Neurological Institute T1 template in SPM by linear and non-linear deformations. The normalisation parameters were then applied to all spatiotemporally realigned functional images, the mean image and structural images, prior to spatial smoothing of fMRI data with an isotropic Gaussian kernel FWHM 10 mm.

First level Statistical Parametric Modelling for each subject used a general linear model with one regressor representing the presentation of a trial (of any type), subject to parametric modulation according to the two conditions “specified” and “chosen,” and also to reaction time. Additional subject specific regressors were included for scans which exceeded threshold for scan to scan movement or variance. This removes the effect of that scan on estimation or parameters for effects of interest (“nulling scans”) (Lemieux et al., 2007; Rowe et al., 2008b,c). A second level model (random effects) was made for each contrast of interest using an ANOVA of the contrast images from each older subject at the first level (patient and older controls). Second level contrasts included “Task,” collapsing “chosen” and “specified trials,” and “Selection,” contrasting chosen with specified actions. The second level model was a pseudofactorial design with group as a between subjects factor (control vs. patient) and treatment as a within subject factor (patients: dopaminergic treatment vs. dopaminergic withdrawal; controls: random assignment of the 2 sessions in the same procedure as randomisation of the order of patient treatment but for controls no medication was given).

### Dynamic causal modelling

**Fig. 1:**
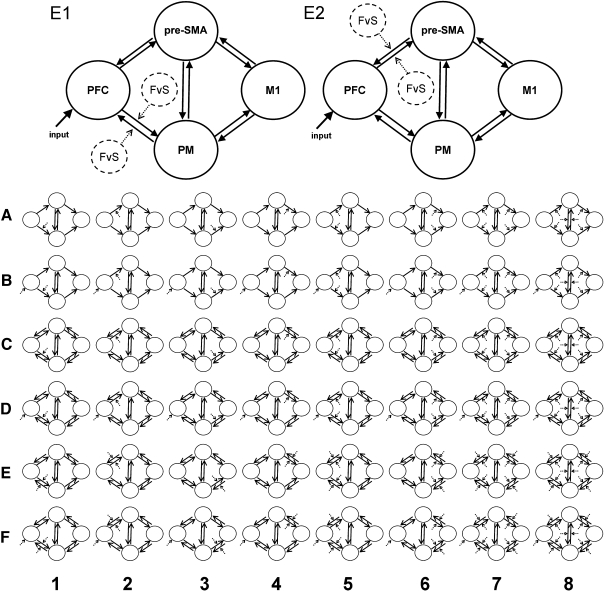
Schematic representations of the 48 models compared in this study. Models are illustrated in sets, grouped by intrinsic connectivity structure (letters A–F) and by the modulation of connectivity by the difference between chosen and specified conditions (numbers 1–8). Models E1 and E2 are enlarged. The free energy estimate of the log evidence, *F*, is greatest for model E2 in young and old healthy subjects, and patients on dopaminergic therapy. Model E1 is more likely in patients in a relative “off” state induced by medication withdrawal. The models differ in the contextual modulation of task related connectivity, which is manifest in changing connectivity of prefrontal to pre-SMA connectivity (E2) or prefrontal to premotor cortex connectivity (E1). PFC = prefrontal cortex, pre-SMA = pre-supplementary motor area, PM = premotor cortex, M1 = primary motor cortex. For clarity, the labels are omitted from the small model illustrations. The contextual modulation is illustrated by a dotted arrow.

The DCM proceeded with the following schema: (1) define an anatomical network of contributory regions, (2) define a set of models based on variations of intrinsic connections within this network, principally by specifying unidirectional or bidirectional connectivity (see Fig. 1), (3) specify bilinear terms, indicating the modulatory effect of task type (chosen vs. specified trials), (4) specify direct inputs to prefrontal cortex, that in every model was the onset of the go stimulus, and in a subset of models also the task type (chosen vs. specified), (5) extract BOLD fMRI time series from the network regions for each subject, (6) estimate the models, (7) compare all models using the free energy estimate of the model evidence, grouping according to age, disease or treatment, (8) compare the leading models, in terms of relevant between-subjects factors, using fixed and random effects methods described below.

The anatomical network model of frontal cortical motor interactions was based on our previous model for structural equation modelling of action in Parkinson's disease (Rowe et al., 2002b). It included left dorsolateral prefrontal cortex (PFC), left lateral premotor cortex (PM), pre-supplementary motor area (pre-SMA) and left primary motor cortex (M1). The coordinates of these regions were based on local peaks in the second level model of regional activations (see supplementary Table S2) BOLD fMRI time series were extracted from regions 10 mm in diameter centred on these locations, from each subject, using the first eigenvariate of voxels above a subject specific *F*-threshold of *p* < 0.001 (uncorrected). Where a subject had no voxel above threshold at the specified location, the centre of the ROI was adjusted to the nearest suprathreshold voxel, and checked to ensure that it lay within an appropriate anatomical range, for example, that the motor cortical region of interest lay on the precentral bank of the central sulcus. Table S2 shows the second level contrast coordinates, and the mean of actual coordinates used by all subjects as the centres of regions for time series extraction.

The connections between these regions were specified as intrinsic connections (matrix A in DCM) as shown in Fig. 1. The initiation of an action, whether specified or chosen, was modelled as a task input to prefrontal cortex (matrix C in DCM). This reflected the rostro-caudal sequence of frontal cortical activation in neurophysiological measurements of cued manual actions (Nishitani and Hari, 2000; Pedersen et al., 1998). DCM enables the comparison of non-nested models for the same data. We therefore specified 48 variations on this model, based on the presence of unidirectional (Fig. 1 model sets A and B) or bidirectional connections between these cortical regions (Fig. 1 model sets C–F).

The difference between the two conditions (chosen vs. specified) may manifest itself through changes in cortico-cortical connectivity, constituting a psychophysiological interaction (PPI) (Friston et al., 1997). Here, the context of chosen versus specified actions is proposed to be mediated by a change in the intrinsic connectivity of the motor network, consequent to transient perturbation of that network by the stimulus. Such psychophysiological interactions are implemented as bilinear terms in DCM (matrix B) (Friston et al., 2003; Penny et al., 2004a), in contrast to the moderator variables in structural equation models (Rowe et al., 2002a,b, 2005) or interaction regressors in general linear models of PPIs.

The set of models varied systematically in terms of the connections which were subject to contextual modulation. For example, was the difference between trial types manifest as differences in connectivity between PFC and PMC or between PFC and pre-SMA? See Fig. 1 for the full set of models. In general, the model sets included modulation of each connection separately (Fig. 1 model sets 1–4), or rostral connections (Fig. 1 model set 5), caudal connections (Fig. 1 model set 6), both rostral and caudal connections and all connections including between premotor cortex and pre-SMA (Fig. 1 model sets 7 and 8). These patterns of modulation were applied to rostro-caudal and reciprocal connections when these existed in the model. In addition, the difference between selection and specified conditions was included as a direct influence on prefrontal cortex in a subset of models (Fig. 1, model sets B, D, and F).

The dynamic causal model was implemented for each session, for each subject, for each model (4320 in total). Each model was fitted by SPM5, with expectation maximisation procedure for estimation of the parameters of intrinsic, bilinear and input connections, and the parameters of the biophysical model of the evoked haemodynamic response to neural activation (Friston et al., 2003).

In addition to the model specific estimates of the connection parameters, each estimation procedure also derives an estimated bound on the model evidence, using the free-energy criterion, *F* (Friston et al., 2007). This free-energy criterion indicates the accuracy of the model (as log-likelihood of the data) corrected for the complexity of the model. The complexity term depends on both the number of parameters and the dependencies between parameters. Unlike previous approximations of the model evidence, such as Akaike's information criterion and Bayes' information criterion (Penny et al., 2004a), the free-energy estimate adjusts the penalty for model complexity according to both *a priori* and *a posteriori* independence of the model parameters. We therefore used the negative free energy estimates to compare models within and between subjects.

The negative free energy estimate can be summed over subjects for each model to estimate the cumulative evidence for that group of subjects, comparable to the use of Group Bayes factors, This approach has been used for fMRI (Acs and Greenlee, 2008; Allen et al., 2008 ; Grol et al., 2007; Heim et al., 2009; Kumar et al., 2007; Leff et al., 2008; Smith et al., 2006; Stephan et al., 2007a,b; Summerfield and Koechlin, 2008) and EEG data (Garrido et al., 2008, 2009, 2007). This approach is also termed a fixed effects method, because it assumes that all members of the groups generated data from the same underlying model, without the model itself being a random variable between subjects.

**Fig. 2:**
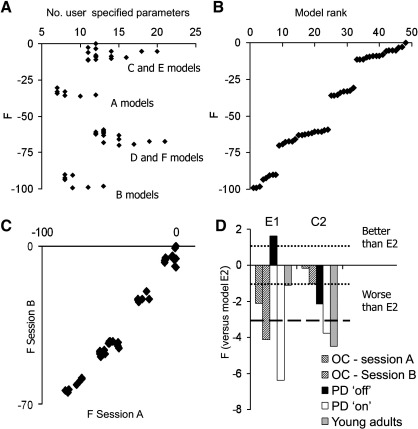
(A) For all healthy subjects the difference in log-evidence, estimated by the free energy *F* for each model, is plotted against the number of user specified connection parameters in the models. For each set of structural models (labelled A–F, according to Fig. 1) there are further differences in *F* according to the bilinear effects representing contextual modulation of connectivity. The most likely model of all E2 is referenced at *F* = 0. (B) The log-evidence estimate *F* is plotted against the model rank over all healthy subjects, from least likely = 1 to most likely = 48. (C) The 48 model evidences summed over 15 elderly control subjects on each of two sessions A and B, indicating a strong positive correlation. Evidences are plotted relative to the most likely model, E2. (D) The group differences in log-evidence estimates *F* for models E1 and C2 are plotted relative to the overall preferred model E2. Bars represent the difference between models, for each group by session. For older control subjects (OC) the two sessions were randomly assigned to sessions A and B, matched for first and second sessions. Similarly, for patients with PD, one session was in a relative “off” state induced by medication withdrawal, while the other session was in a relative “on” state with usual medication. Horizontal lines indicate thresholds for positive evidence (dotted) or strong evidence (dashed) in favour of model E2 (if below the line) or against model E2 (if above the line).

We undertook several approaches to assess the reproducibility of DCM model selection. First, we asked whether the most likely model over all healthy subjects is also separately identified in two separate groups of healthy adults. Second, we assessed whether the same model is identified from a group of subjects when the same experiment is repeated on two separate sessions. For this, we present the model rankings based on the group sum of log-evidence (*F*) (Fig. 2).

The selection of one or other model as most likely depends on the difference in log-evidence, which can also be expressed in terms of the Bayes factor. Given models *A* and *B*, the Bayes factor (BF) comparing model *A* to model *B* is defined as the ratio of model evidences (Kass and Raftery, 1995; Raftery, 1995) such that the log-BF corresponds to the difference in log-evidences. By convention (Raftery, 1995) BF in the range 3–20 indicates positive evidence for model A over B; BF in the range 20–150 indicates strong evidence; and BF > 150 indicates very strong evidence. The equivalent thresholds for differences in log-evidences are 1.1–3, 3–5 and > 5 respectively. The approach enables the comparison of non-nested models (Fig. 2D).

Stephan et al. (2009a,b) have recently shown that model selection methods that adopt a fixed effects model (using Group Bayes Factors of sum of group log-evidences as above) can be adversely affected by outliers (Stephan et al., 2009a). They introduced a hierarchical Bayesian model selection procedure which accommodates group heterogeneity, estimating the probabilities of a set of models (with a Dirichlet distribution) and the probability of a subject's data given each model. This does not assume that all subjects in a group generated their data according to the same model.

The hierarchical Bayesian model selection approach is equivalent to a random effects analysis incorporating between subject differences in the probability of model having generated the group data. For the current study, this procedure was implemented within SPM8, using initially all 48 models, then repeated using the three most likely models as above for clarity of comparison. This procedure was repeated for healthy older controls and PD patients “on” and “off” medication.

Results of this Bayesian model selection procedure for a group can be presented as (a) the expected likelihood of obtaining a given model for any randomly selected subject in the group, or (b) the exceedance probability, which is the belief that a particular model is more likely than any other model (of all models tested), given the group data. Both indices are based on the parameters of the Dirichlet distribution of the models themselves, and therefore the model rankings are the same for both presentations. Both indices sum to one, over all models compared, and may show that one model is more likely for the group data, incorporating the distribution of model probabilities across the group.

Having identified the most likely model, we then explored the parameters within that optimal model, using parametric and non-parametric classical (frequentist) statistics. The use of parametric statistics to assess model parameters has been made in previous DCM studies of condition or groups effects but the values may violate the assumptions of normal distributions, and non-parametric methods are therefore also used. The parameters we examined included the connection weights for intrinsic connectivity (matrix **A**); the connection weights for the bilinear terms expressing the psychophysiological interactions (matrix **B**); the connection weights for direct inputs (matrix **C**), and the ratio of the bilinear term to the intrinsic connection weight (ratio B/A, for non-zero B). Using these values, we compared (1) young vs. older healthy groups, (2) older subjects during sessions, randomly assigned to session A and session B to counterbalance order, rather than first and second session, (3) older subjects vs. patients with PD, (4) patients with PD, “on” and “off” their dopaminergic medication.

As the parameter estimates were not reliable across sessions, we explored the posterior covariances among parameters for intrinsic connections (DCM.A matrix), bilinear terms (DCM.B matrix) and the driving input (DCM.C matrix). We normalised the posterior covariance matrices (output matrix DCM.Cp) for each subject/group/session to get correlation matrices for ease of interpretation. We calculated the “group × session” mean correlations for each pair-wise combination of intrinsic connectivity, bilinear term and driving input. For healthy older control subjects we performed 1-sample *t*-tests across the group to test the null hypothesis that the correlations had zero mean, and Bonferroni corrected for multiple comparisons. For the pair-wise correlations among **A**, **B** and **C** matrix parameters, we then correlated these group mean values from two sessions performed by the healthy older controls. Finally, the posterior correlations were in turn compared with the between-sessions reliability of each parameter estimate, to test the hypothesis that greater posterior correlations among parameters would reduce the identifiability and reliability of parameter estimation, and thereby reduce between-sessions correlation of a given parameter.

## Results

### Model selection (fixed effects)

The distribution of log evidences across all models for all healthy subjects is shown in Fig. 2, according to the number of user specified connection parameters, including intrinsic and bilinear terms (Fig. 2A) and the rank of the models (Fig. 2B, from most likely to least likely). It can be seen that models fall into groups, according to model structure, comparing Figs. 2 and 1. Within each group, the addition of more terms, increasing model complexity, does in itself increase the evidence for a model (no positive correlation between *F* and number of parameters). Indeed, the more likely models (E2, E1, C2) are among the simpler models within each set.

Model E2 emerges as the most likely of the 48 models, in healthy subjects. Model C2 is second, and model E1 is third, but the difference in log-evidences, Δ*F*, are − 2.64 and − 3.4 respectively, indicating that there is strong evidence in favour of model E2. There is very strong evidence (Δ*F* > 5, BF > 150) for these three models ahead of the fourth and subsequent models.

In Fig. 2c, one can see the strong positive correlation between the log-evidences *F* across two sessions (spearman rho = 0.96, *n* = 48, *p* < 0.001) indicating that a model that is more likely on one session in healthy controls is also more likely on another session for the same subjects performing the same paradigm. However, many of these models are very unlikely.

Focussing on the three leading models from the whole group of healthy controls, C2, E1 and E2, we now consider the question of re-identification of models, and sensitivity to disease and therapy. Fig. 2D shows the relative differences between models in different sessions within each group. For healthy control subjects, model E2 is the most likely model on both sessions, but there is a variable level of evidence that model E2 is superior. For example, there is positive evidence that E2 is a better model than E1 in both sessions of healthy controls. However, model C2 is not sufficiently worse than E2 to pass the conventional threshold indicating positive evidence in favour of E2. From session A alone in healthy controls, the data do not provide positive or strong evidence that E2 is superior.

Session differences are also clearly seen in the patients, but this session difference is characterised by withdrawal of dopaminergic medication. Whether patients are “on” or “off” medication, model E2 is more likely than model C2. When on medication, model E2 remains more likely than model E1, as for healthy subjects. However, withdrawal of dopaminergic medication leads to a change in the most likely network model, from E2 to E1. The difference between these models is that the bilinear term, expressing the modulation of connectivity under different task conditions, affects interactions between prefrontal and premotor cortex rather than prefrontal cortex and pre-SMA (see Fig. 1 models E1 and E2).

### Bayesian model selection (random effects)

**Fig. 3:**
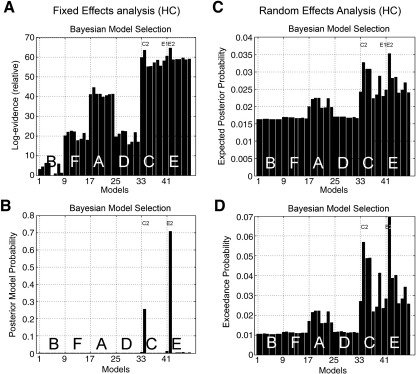
Random effects and fixed effects approaches to group model selection. For a fixed effects analysis of the 48 models (in six sets A to F, see also Fig. 1) the models can be compared using (A) the sum of log-evidences (cf. the Group Bayes Factor for the model) or (B) the equivalent posterior model probability. The leading three models from the groups analysis are E2 > C2 > E1. A random effects analysis using a hierarchical Bayesian model selection procedure (see Methods) over all 48 models compares models in terms of (C) the expected posterior probability that a given subject had generated data according to a given model or (D) the exceedance probability representing the confidence that a given model is more likely than any other model.

**Fig. 4:**
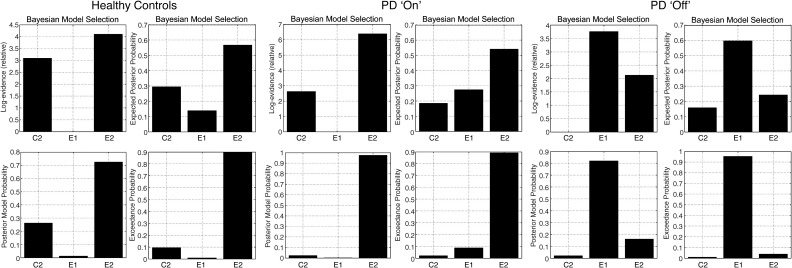
Random effects and Fixed effects Bayesian Model Selection approaches to group model selection for Healthy older controls, PD patients “on” and PD patients “off,” for the leading models E2, E1 and C2. Fixed effects analyses results are presented as log-evidences and Posterior Model Probability (PMP). Random effects analyses are presented as Expected Posterior Probability (EPP) or Exceedance Probabilities (ExPr). It can be seen that by both approaches, model E2 is preferred in Healthy Controls and PD “on” patients, but that model E1 is preferred in PD “off” patients. The difference between fixed and random effects models is seen for PD “on” patients, for whom model E1 is second most likely by the random effects method, but very unlikely by the fixed effects method.

Among healthy controls, the random effects method of Bayesian model selection again identified model E2 as most likely. A comparison of the fixed effects and random effects approaches is shown in Figs. 3 and 4. The results first confirm the clustering of models in to families of structural similarity. For the three more likely models (E2, E1, C2) the random effects and fixed effects approaches produce similar rankings, but the differences between models are easily appreciated with the random effects approach. Considering the three most likely models in Fig. 4, for any given healthy subject, one would be ∼ 57% likely to identify model E2 as the generator of the data, and for the group as a whole, one is 90% confident that E2 is the most likely model that generated subjects' data.

For patients “on” medication, the group fixed effects and random effects results of model selection are shown in Fig. 4. Again model E2 was most likely to have generated the data for the group, using both random and fixed effects methods. However, the two methods of model selection differ in ranking the next models. The random effects models suggest that model E1 was second most likely to have generated the data for “on” PD patients. The difference may be explained in terms of a subset of PD “on” patients with features of PD “off” patients (see next paragraph). This subset did not have sufficiently marked model evidences individually to influence the fixed effects analysis (in which model E1 was much less likely). An alternative interpretation is that a subset of PD “on” patients were characterised by normal behaviours, with strong individual model evidences, increasing the similarity of the fixed effects analysis for PD “on” and health aged controls.

Patients in a relative “off” state after medication withdrawal are qualitatively different. As shown in Fig. 4, it was more likely that model E1 generated their data, a model in which selection to action is associated with increased coupling between prefrontal cortex and lateral premotor regions but not pre-SMA. Here, both fixed and random effects methods were concordant, providing > 90% posterior model probability or > 95% exceedance probability in favour of model E1.

### Parameter estimation

The effect of direct trial input to the prefrontal cortex (the ‘injection’ of perturbation into the network, DCM matrix C) was positive in all groups (older subjects mean 0.10 Hz SE 0.02, younger subjects mean 0.09 Hz SE 0.02). Values from the older subjects were entered into a 2 × 2 repeated measures ANOVA (factor 1: group, Older controls vs. PD patients; factor 2 drug treatment/session) which indicated no differences between groups or sessions, or group by session interaction (all *F*_(1,14)_ < 1, ns). This implies that neither PD nor dopaminergic medication alters the parameter estimate for the driving input.

There were bidirectional intrinsic connections between nodes in the most likely model E2 (ten intrinsic connections, DCM matrix A). These were entered into a 10 × 2 × 2 repeated measures ANOVA (factor 1: group, Older controls vs. PD patients; factor 2 drug treatment/session; factor 3 connection). There was evidence of strong differences in intrinsic connectivity for the different connections (*F*_(9,117) _= 24, *p* < 0.001) but no group difference (*F*_(1,13) _= 2.9, ns) or any other main effect, 2-way and 3-way interactions (*F* < 1.4, ns). Thus, the intrinsic parameters are not significantly different between groups, and none of the connections was consistently higher or lower on different sessions, even when the sessions were associated with drug differences in the PD group.

**Fig. 5:**
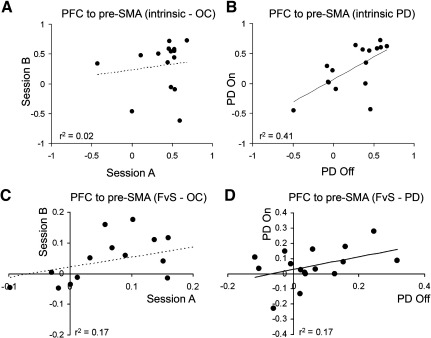
The estimates of intrinsic connectivity PFC → pre-SMA across two sessions was poorly correlated in healthy older controls (OC, panel A) but positively correlated in patients with Parkinson's disease (PD, panel B). The estimate of the bilinear term in older healthy control subjects (C) and PD patients (D), each group repeating the same paradigm on two occasions. These estimates of the bilinear term express the modulation of PFC → pre-SMA connectivity by trial context (chosen vs. specified response trials). The parametric correlations are not significant, but see also Results for non-parametric statistics.

For healthy control subjects, the correlation between intrinsic parameters across two sessions was also not high e.g. for the PFC → pre-SMA connection, *r*^2 ^= 0.02 (see Fig. 5A, ns), with other connections ranging *r*^2^ between 0.0 and 0.13. For PD patients however, correlations were higher, e.g. for PFC → pre-SMA *r*^2 ^= 0.41 (see Fig. 5B, *n* = 16, *p* < 0.01), with other connections varying in terms of *r*^2^, between 0.04 and 0.26 (all ns) except for SMA → MC (*r*^2 ^= 0.48, *p* < 0.01) and pre-SMA → PM (*r*^2 ^= 0.39, *p* < 0.05). Despite these low correlations in the value of the intrinsic connectivity parameters, the sign or direction of effect is well preserved i.e. a positive connection on one session remains positive on a second occasion. For five of ten intrinsic connections, no subject altered sign. For one connection the sign was reversed in one subject only. For the four other intrinsic connections, four or five out of 15 subjects changed sign between the two sessions and one cannot therefore reject the null hypothesis of random sign for these connections (PFC → PM, PFC → pre-SMA, PM → PFC, pre-SMA → PFC; chi-squared 1.7–3.3, *df* 1, ns).

The bilinear terms are generally the main interest in DCM studies of effective connectivity, expressing a psychophysiological interaction. The preferred model E2 included bilinear modulation of connectivity of PFC → pre-SMA (mean of older subjects = 0.06, SE = 0.01) and vice versa (mean of older subjects = 0.05, SE = 0.01). The values of the bilinear term from each subject and session was entered into a 2 × 2 × 2 repeated measures ANOVA (factor 1: group, Older controls vs. PD patients; factor 2 drug treatment/session; factor 3 connection, PFC → pre-SMA vs. pre-SMA → PFC). All main effects, 2-way and 3-way interactions were clearly negative (*F*_(1,14)_ < 1, ns) except for the weak effect for a group by drug interaction (*F*_(1,14)_ = 2.2, *p* = 0.13). One-sample Kolmogorov–Smirnov tests of the distribution of parameters for each of the two connections for each drug and group (8 tests, K-S *z* < 1, *N* = 15 or 16, each ns) or for the two connections pooled over all older subjects-sessions (2 tests: K-S *z* < 1.3, *N* = 62, both ns) did not reveal any significant deviations from normality.

The bilinear terms expressing contextual modulation (chosen vs. specified) of response correlated weakly across healthy older subjects between sessions for the modulation of PFC → pre-SMA connectivity, Pearson *r*^2 ^= 0.17, *n* = 15, *p* = 0.06, although non-parametric Spearman rho did reach significance threshold (see Fig. 5C–D, rho = 0.504, *n* = 15, *p* = 0.03). Similar between sessions correlations for this connection were seen for PD patients (Fig. 5D). For the reverse connection, pre-SMA → PFC, the correlation was not significant (*r*^2 ^= 0.01, *n* = 15, ns; rho = 0.02).

One may be more interested in the sign of a psychophysiological interaction than its magnitude i.e. does the context increase or decrease connectivity? In healthy controls, 11/15 subjects had the same sign on both sessions, and one cannot reject the null hypothesis that the direction of effect is not preserved over repeat sessions (chi-squared = 3.3, *df* 1, *p* = 0.07). Among patients, 11/16 subjects maintained sign and for this group (with drug treatment) one cannot reject the null hypothesis that the direction of effect is not preserved over repeat sessions with drug withdrawal (chi-squared 2.3, *df* 1, ns).

### Posterior covariances and identifiability of parameters

**Fig. 6:**
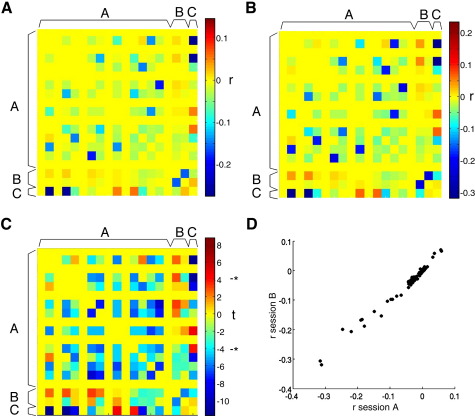
The posterior covariances among parameter estimates (output matrix DCM.Cp) have been normalised to correlation matrices, and averaged across healthy older control subjects (A) and patients with PD (B). The matrices show the group averaged posterior correlations among intrinsic connections represented by the DCM.A matrix “A,” the two bilinear effects from DCM.B “B” and the direct driving inputs from DCM.C “C.” At a glance, one can see the prevalence of non-zero correlations between connectivity parameters, and the similarity of these posterior covariances in the two groups. (C) The map of *t*-statistics from 2-tailed one-sample *t*-tests at each element of the posterior correlation matrix for healthy subjects. The absolute values of correlations in panels (A) and (B) were small (typically less than ± 0.1 and all less than ± 0.3) but these interdependencies are significantly different from zero across the group (C). The asterisk indicates *t*-threshold for significance (*p* < 0.05, Bonferroni corrected for multiple tests). In panel (D) we show that the averaged values in the posterior correlations matrices from a group of older healthy subjects are reproduced on two sessions for the preferred model (E2) (Spearmans' rho = 0.99) and that these values are mostly negative (61/78 = 78%).

One of the factors that determine the reliability of parameter estimation is identifiability of parameters. Pronounced posterior covariances among parameters, indicating inter-dependencies, make the parameters difficult to identify (Deneux and Faugeras, 2006; Stephan et al., 2009b, 2007b). In our optimal model E2, and the nearest models E1 and C2, there were significant posterior covariances among the parameters for intrinsic, bilinear and driving inputs. We have normalised these values (from the output DCM.Cp) and illustrate them in Fig. 6 for model E2.

In both healthy older controls (Fig. 6A) and patients with PD (Fig. 6B) there are similar patterns of posterior correlations. The majority of correlations were negative (61/78 = 78%). Although the correlations are generally small (most less than ± 0.1, with a few up to − 0.3) they are numerous and highly consistent for this model (Figs. 6C, D) both within a session and between sessions.

We looked for a correlation between the inter-session correlations of intrinsic parameters between two sessions, and the posterior correlations among connections. It was predicted that for any given connection, higher maximal posterior correlations or higher sum of posterior correlations (real or absolute) would themselves correlate negatively with the intersession reliability of the connection's parameter estimate. For older controls, no such relationship was found (all *r* < 0.15, *p* > 0.1). This negative result is perhaps because a different subset of posterior correlations was relevant, or because of complex interactions among them.

## Discussion

### Model selection and parameter estimation

Our results provide clear evidence that a DCM model selection procedures are able to re-identify the most likely model from a group of subjects performing the same task on two separate occasions. This model was also most likely when considering the evidence over a larger group of old and young healthy adults. When each session was considered separately, there was not sufficient evidence to select the most likely model (E2) over a second model (C2). However, model C2 was nested within model E2, differing only by the modulation of the feedback as well as feedforward connections. These two models were similar to each other and significantly separated in terms of model-evidence from all other models. For identification of the most likely model (E2), both fixed and random effects modelling of the group data were concordant for healthy subjects and “on” PD patients.

Importantly for the future application of DCM to clinical and pharmacological studies, DCM model selection did identify a different model (E1) as most likely in patients “off” mediation. Model E1 was more likely than any other model in “off” patients, while the normal model E2 was more likely in patients “on” medication. For some studies, exploring the effects of disease or drug on network connectivity, it would be sufficient to stop at this stage of model selection, with strong evidence for the inference that the disease or drug therapy had changed network connectivity from one pattern to another.

In our paradigm and groups, it is noteworthy that the DCM model selection confirms a shift in the causal interactions underlying response selection, away from a medial prefrontal-SMA route towards a lateral prefrontal-premotor. This accords with the earlier evidence of a functional deafferentation of the pre-SMA and SMA (Dick et al., 1986; Rowe et al., 2002b) and a greater role for lateral premotor cortex in patients making complex finger movements (Samuel et al., 1997).

Although it may be sufficient to stop with evidence of alternative network models, it is common practice to analyse the parameter estimates within these models. This is often motivated by the aims of scientific transparency and artefact detection, as well as the theoretical importance of parameter estimates for some research questions (Summerfield et al., 2006). In addition, secondary analysis of parameter estimates using frequentist methods like *t*-tests or ANOVA have been used to compare groups, or correlate with between-subject measures (Passamonti et al., 2008; Sonty et al., 2007). This is appropriate for group studies with some caveats (Stephan et al., 2009b) and it is possible therefore to use connectivity parameters to compare two groups or to compare connectivity under two therapeutic conditions.

Our results suggest caution when using this parameter based approach, at least for some models. Whereas Schuyler et al. (2009) found excellent reliability across sessions for DCM connectivity parameters, this was not the case for the preferred model in the current study. Although many intrinsic connections did maintain their sign over two sessions in all subjects, four out of ten intrinsic connections changed sign in a third of healthy participants, when *a priori* no change would be expected. Parametric correlations were poor for intrinsic connections and bilinear terms across sessions in healthy subjects.

For patients, significant positive correlations between parameter estimates for the two sessions were observed, but only for connections of the pre-SMA: with prefrontal, motor and premotor cortex. Since the pre-SMA and SMA are especially vulnerable cortical regions to Parkinson's disease (Haslinger et al., 2001; Jahanshahi et al., 1995; Playford et al., 1992; Sabatini et al., 2000; Samuel et al., 1997; Yu et al., 2007) our subjects at different stages of PD will have had a higher within-group between-subjects variance of pre-SMA/SMA connectivity than healthy control subjects. This high between subjects variance in pre-SMA function may have increased the signal to noise for correlations across separate sessions, despite drug treatment in one session.

Why is the reliability of parameter estimates so poor, and in contrast to previous studies (Schuyler et al., 2009) and the reliability of model selection? One important factor is the posterior covariances among parameters e.g. if a model allowed one parameter to compensate for changes in another parameter, maintaining overall model evidence, then the anti-correlated values of both parameters could vary widely from session to session. This lack of identifiability of the parameters limits reliability across sessions, affecting both hemodynamic (Deneux and Faugeras, 2006) and connectivity parameters (Stephan et al., 2009b, 2007b) in second level statistical tests on these parameters (but not model selection). When posterior covariances among connectivity parameters are expressed as correlations for our preferred model E2 the values seem low (generally 0 to − 0.1; Fig. 6). However, they were consistent across sessions and across groups.

These posterior covariances will be model specific, leading to the possibility that a better model (by model evidence) has worse reliability of parameter estimates. Within a preferred model, one could in principle use regularisation of the intrinsic and bilinear parameters, or re-estimate the models after constraining strongly covarying parameters. This approach might improve the between sessions reproducibility of parameter estimates, but it is not yet developed. Model complexity may influence the reliability of parameter estimation. The high reliability reported by Schuyler et al. (2009) refers to structurally very simple models, with two or three regions connected by feed-forward only connections. In contrast, our Bayesian model selection identified a model of intermediate complexity (E2) which included feed-forward and feed-back connections. For our study and model, the presence of significant posterior dependencies among parameter estimates is an indication for caution before using secondary (frequentist) statistics applied to the parameter estimates.

As within-subject variance sets a minimum variance for between subjects comparisons, the poor reliability increases the chance of failing to observe group differences in terms of estimated parameters. While this does not invalidate previous reports of group effects on parameter estimates (Passamonti et al., 2008; Rowe et al., 2008b; Sonty et al., 2007; Summerfield et al., 2006) it does increase the risk of type II error in future studies. We recommend that if a hypothesis is to be tested by second level tests on parameters, then the posterior covariance matrix is inspected closely. For single group studies, Bayesian parameter averaging (Garrido et al., 2007) may be used as an alternative, weighting the group's joint posterior distribution by the precisions of each subject and accounting for the posterior covariances among parameters. For group studies, new methods for Bayesian Model Averaging are awaited.

There are other possible reasons for the difference between our reliability estimates and these reported by Schuyler et al. (2009) for fMRI-based DCM. Both studies used relatively simple sensori-motor paradigms and conventional data pre-processing. However, the interval between sessions differed: 5 min for Schuyler et al. (2009) and weeks for this study. The longer interval may increase differences in cognitive strategies, haemodynamic properties and neuronal dynamics within distributed networks.

Another potential confound is differential movement during sessions, either between groups (e.g. PD versus healthy subjects) or within subjects (e.g. on- versus off-medication). We used a ‘scan nulling’ approach with standard thresholds across all subjects (Lemieux et al., 2007; Rowe et al., 2008b,c) to correct for movements in first level models and time-series extraction. PD patients tended to make more such movements. However, reliability was poor even among healthy controls with few significant movements.

### Model space

If model selection procedures are sufficient and more robust, one must consider how many models should be compared. The answer must be theoretically motivated for each study, although there may be constraints on the upper limit imposed by current technology and time available to compute and compare a very large set of models. For example, for our four-node anatomic network and paradigm, there are approximately 10^10^ models that could be compared, most of which are *a priori* not plausible. Some studies have used a single model and emphasised the parameterisation within this model (Summerfield et al., 2006). Typically however, a small set of models, 2 < *n* < 15, have been compared in published studies to date. These models have been chosen not because they enable an exhaustive search of model space. The model set has been chosen because the differences between models represent theoretically relevant alternative hypotheses about which connections are subject to contextual modulation (Acs and Greenlee, 2008; den Ouden et al., 2009) or the structure of intrinsic connections including presence or absence of feedback connections and the roles of direct versus indirect regional interactions (den Ouden et al., 2009; Fairhall and Ishai, 2007; Stephan et al., 2007a).

This study used a relatively large set of models in relation to the published literature (*n* = 48). There were several reasons for using a larger model set. First, we wished to include a basic model that was structurally similar to that used for SEM in a previous study, even though this was likely to be a poor representation of the underlying network because of the absence of feedback connections. Second, we wanted to compare models with and without feedback, rather than assuming only a rostro-caudal cascade of influence. Third, we wanted to include perturbation of the PFC by both task and the contextual moderator in some models. In addition to modulation of downstream activity by contextual modulation of connectivity, the inputs to the network via prefrontal cortex might also have been varied between conditions. Fourth, we wanted to explore the likely sites of contextual modulation of connectivity, particularly in relation to the functional roles of the pre-SMA and premotor cortex in Parkinson's disease.

These different objectives necessitated a large set of models. Our set of models also allowed an empirical assessment of the performance of the model complexity term including the free energy estimate of the log-evidence for each mode. This complexity term is the Kullback–Leibler divergence between the posterior and prior density (Friston et al., 2007) that includes the interdependencies between model parameters. Simply adding more user specified parameters e.g. intrinsic or bilinear effects in the model, does not necessarily improve model goodness, even if the model becomes more accurate. The model evidence decreases if one makes bad assumptions about the model structure and parameter distributions. From Figs. 1 and 2a, it can be seen that more complex sets of models (sets D and F vs. B) may actually worsen model evidence, and that even within a model set additional bilinear terms (sets 1 to 6) may also worsen the overall model evidence, unless of course they represent the “real” modulatory effects within the generative brain network.

There are several limitations to our study. We may have lacked power to detect underlying correlations of connectivity parameters across sessions. The size of our groups are within the typical range for classical statistical parametric mapping of regional activations (SPM) and adequately powered for visuomotor tasks like the one used in this study (Thirion et al., 2007) (supported for our chosen task by the concordance over many studies, see Introduction). Although our data indicate DCM based model selection does re-identify the most likely model on two occasions in healthy adults, power estimates for DCM are not yet widely available (Goulden et al., 2009).

Our study used standard BOLD-EPI acquisition sequences and a commonly used pre-processing pathway for fMRI data intended for both DCM and classical analysis. fMRI may be confounded by session effects (McGonigle et al., 2000) due to many factors including variable BOLD sensitivity, differential motion artefacts (Lund et al., 2005) and potential confounds introduced by drug effects on neurovascular coupling and the BOLD signal (Iannetti and Wise, 2007). These factors may also affect analyses of effective connectivity, including DCM, and diminish the measured correlations of connectivity parameters over sessions. However, the subject-specific and region-specific estimation of the parameters of the forward model (from neuronal activity to BOLD signal) enables a wide variation in the shape and timing of the evoked haemodynamic response. Moreover, the haemodynamic parameters defining this forward model remain constant over all task conditions within a session, such that the approximation to the true evoked haemodynamic response to neural activity is applicable to all event types. Therefore, confounding effects of drug and sessional differences in BOLD might be less for DCM based estimates of anatomically defined effective connectivity changes driven by experimental factors, than estimates of whole brain functional connectivity.

## Conclusion

Dynamic Causal Modelling model selection procedures are able to re-identify the same model as most likely, given independent data acquired from a group of subjects on 2 separate days. A different model was identified as most likely in patients with Parkinson's disease after medication withdrawal, revealing a switch from a medial to lateral cortical route for voluntary actions in the frontal lobe. Both random effects and fixed effects approaches are practical to compare many models across and between large groups, but model selection was more robust than parameter estimation within the optimal model for healthy controls. Our results support the future use of DCM in clinical and pharmacological fMRI studies.

## Appendix A Supplementary data

Supplementary Table S1 Summary statistics for the PD patients and the older (OC) and younger participants.

Supplementary Table S2 Coordinates used for extraction of time series. The coordinates may vary slightly across individuals from the group mean coordinate because of individual variation in the location and magnitude of activations.
