# Supplementary material for: Dynamic causal modelling of effective connectivity from fMRI: Are results reproducible and sensitive to Parkinson's disease and its treatment?
Source: Neuroimage. 2010 Sep;52(3):1015–26. doi: 10.1016/j.neuroimage.2009.12.080 (PMC3021391; doi:10.1016/j.neuroimage.2009.12.080)
Supplement: Supplementary Table S1 — Summary statistics for the PD patients and the older (OC) and younger participants. [file mmc1.pdf]

Table S1. Summary statistics for the PD patients and the older (OC) and younger participants.

No	Sex	Age	Duration	H+Y (on)	UPDRS		L-dopa	L-dopa equivalent[1]	Other Medication[2]				
					On	Off			A	C	R	E	
1	f	65	4.6	2.5	30	38	600	1560			24		
2	m	61	15.4	2	17	53	2200	2585	300				T 300
3	m	59	5.7	2	29	36	500	1140	200	4			
4	f	53	8.7	2	14	24	900	2200	100		21		
5	m	78	13.5	3	30	50	700	512					O 120
6	f	59	5.5	2	31	34	300	1260		6			
7	m	68	9.0	2	20	32	250	915		4			S 5
8	m	66	6.9	2	25	33	800	1009	200	1		600	
9	m	65	13.5	2.5	19	36	700	1196				800	S 10
10	f	65	5.0	2	7	12	250	1090			21		O 50, D 30
11	f	49	9.5	2	15	34	300	1200	200		15		
12	f	78	5.7	1	1	14	600	600					
13	f	62	5.4	1	10	26	500	1020			18	600	
14	m	64	3.0	2	17	20	400	1290	100				P 2.1
15	m	62	5.2	2	19	30	800	1640	100				P 1
16	m	69	4.8	2	18	29	400	1120		4			S 1.25
**9m, 7f**	**Mean**	**63.9**	**7.6**	**2.0**	**18.9**	**31.3**	**637.5**	**1271.1**					
	Sd	7.5	3.7	0.5	8.6	11.0	465.7	527.8					

**Older Controls[3]**		
**7m, 8f**	**Mean**	**66.5**
	Sd	5.9
**Younger Controls**		
**17m, 11f**	**Mean**	**31.0**
	Sd	10.6

[1] Equivalent levodopa dose = [levodopa (x 1.2 if COMT inhibitor)(x 1.2 if 10 mg of S or x 1.1 if 5 mg of S)] + [P x 400] + [R x 40] + [C x 160] + [pergolide x 200] + [bromocriptine x 10] + [lisuride x 160]; all doses are in milligrams. (Williams Gray C. (2007) J Neurosci 27 (18) 4832-4838).

[2] Other drugs as mg/day are: A= amantadine, C= Cabergoline, R = Ropinirole, E = entacapone, T= tolcapone, S = selegiline, P = pramipexole, O = orphenadrine, D = domperidone.

[3] Medications taken by four older controls were: 1 x fenofibrate, 2 x antihypertensives, 1 x ibuprofen, co-codamol, zopiclone, tamsulosin hydrochloride and etodolac.

Duration = Duration of disease from diagnosis in years. H+Y = Hoehn and Yahr rating scale; UPDRS = Unified Parkinson’s Disease Rating Scale motor subscale III
