# Supplementary material for: Dynamic causal modelling of effective connectivity from fMRI: Are results reproducible and sensitive to Parkinson's disease and its treatment?
Source: Neuroimage. 2010 Sep;52(3):1015–26. doi: 10.1016/j.neuroimage.2009.12.080 (PMC3021391; doi:10.1016/j.neuroimage.2009.12.080)
Supplement: Supplementary Table S2 — Coordinates used for extraction of time series. The coordinates may vary slightly across individuals from the group mean coordinate because of individual variation in the location and magnitude of activations. [file mmc2.pdf]

Supplementary Table S2. Coordinates used for extraction of time series. The coordinates may vary slightly across individuals from the group mean coordinate because of individual variation in the location and magnitude of activations.

		Coordinates used for time series extraction			Second level group analysis coordinates		
		x	y	z	x	y	z
Prefrontal cortex	Mean	-40.16	37.69	24.04	-40	38	24
	sd	3.99	4.67	5.19			
Premotor Cortex	Mean	-30.49	-8.31	52.40	-30	-8	52
	sd	3.23	2.16	3.26			
Primary motor cortex	Mean	-37.84	-19.93	61.82	-38	-20	62
	sd	1.85	1.42	0.98			
Pre-SMA	Mean	-4.53	2.18	55.96	-4	2	56
	sd	2.86	3.57	3.42			
